# “Is it making any difference?” A qualitative study examining the treatment‐taking experiences of asymptomatic people living with HIV in the context of Treat‐all in Eswatini

**DOI:** 10.1002/jia2.25220

**Published:** 2019-01-29

**Authors:** Shona Horter, Alison Wringe, Zanele Thabede, Velibanti Dlamini, Bernhard Kerschberger, Munyaradzi Pasipamire, Nomthandazo Lukhele, Barbara Rusch, Janet Seeley

**Affiliations:** ^1^ Médecins Sans Frontières Nhlangano Eswatini; ^2^ London School of Hygiene and Tropical Medicine London UK; ^3^ Swaziland National AIDS Programme Ministry of Health Mbabane Eswatini; ^4^ Médecins Sans Frontières Geneva Switzerland

**Keywords:** Treat‐all, HIV, adherence, asymptomatic, qualitative, Swaziland

## Abstract

**Introduction:**

Treat‐all is being implemented in several African settings, in accordance with 2015 World Health Organisation guidelines. The factors known to undermine adherence to antiretroviral therapy (ART) may change in the context of Treat‐all, where people living with HIV (PLHIV) increasingly initiate ART at earlier, asymptomatic stages of disease, soon after diagnosis. This paper aimed to examine the asymptomatic PLHIV's experiences engaging with early ART initiation under the Treat‐all policy, including how they navigate treatment‐taking over the longer term.

**Methods:**

A longitudinal qualitative study was conducted within a Médecins Sans Frontières/Ministry of Health Treat‐all pilot in Shiselweni, southern Eswatini. The Treat‐all pilot began in October 2014, adopted into national policy in October 2016. Participants were recruited purposively to include newly diagnosed, clinically asymptomatic PLHIV with a range of treatment‐taking experiences, and healthcare workers (HCW) with various roles. This analysis drew upon a sub‐sample of 17 PLHIV who had been on ART for at least 12 months, with mean 20 months on ART at first interview, and who undertook three interviews each. Additionally, 20 HCWs were interviewed once. Interviews were conducted from August 2016 to September 2017. Data were analysed thematically using coding, drawing upon principles of grounded theory, and aided by Nvivo 11.

**Results:**

It was important for PLHIV to perceive the need for treatment, and to have evidence of its effectiveness to motivate their treatment‐taking, thereby supporting engagement with care. For some, coming to terms with a HIV diagnosis or re‐interpreting past illnesses as signs of HIV could point to the need for ART to prevent health deterioration and prolong life. However, others doubted the accuracy of an HIV diagnosis and the need for treatment in the absence of symptoms or signs of ill health, with some experimenting with treatment‐taking as a means of seeking evidence of their need for treatment and its effect. Viral load monitoring appeared important in offering a view of the effect of treatment on the level of the virus, thereby motivating continued treatment‐taking.

**Conclusions:**

These findings highlight the importance of PLHIV perceiving need for treatment and having evidence of the difference that ART is making to them for motivating treatment‐taking. Patient support should be adapted to address these concerns, and viral load monitoring made routinely available within Treat‐all care, with communication of suppressed results emphasized to patients.

## 1 Introduction

Treat‐all is being implemented in several African settings, accordant with 2015 World Health Organisation (WHO) guidelines recommending regular HIV testing and immediate offer of antiretroviral therapy (ART) for all those diagnosed HIV‐positive, irrespective of immunological status 1. To contribute towards reduced HIV incidence and the hoped‐for elimination of AIDS, Treat‐all requires engagement of individual people living with HIV (PLHIV) with HIV testing, prompt initiation of ART and continued lifelong treatment 2, 3, 4, 5. However, shortfalls exist across the treatment and care cascade 6, 7, 8.

There is extensive research examining adherence to ART in African settings under previous treatment guidelines, highlighting the individual, social and structural factors influencing engagement with treatment and care among PLHIV 9, 10, 11. Motivation for adherence may be stronger when patients are very sick at ART initiation, as the effects of ART in enabling a return to health and strength can create a sense of need for treatment and belief in its efficacy, and past illness experiences are drawn upon to motivate continued treatment‐taking 12, 13. Many of these factors change in the Treat‐all context, where PLHIV are initiating ART at earlier, asymptomatic stages of disease, where the time between diagnosis and ART initiation may be expedited, and the length of time on ART may eventually be greater than previously.

Evidence from Prevention of Mother to Child Option‐B+ (Option‐B+) suggests retention in care among women on Option‐B+ is lower than among women starting ART for their own health 14, 15, 16. HIV status acceptance, treatment readiness and perceived need for treatment in the absence of symptoms can undermine pregnant and lactating women's retention, with some disengaging from care once their perceived objective of protecting the baby is fulfilled 17, 18, 19. While these findings provide important insights into understanding how asymptomatic PLHIV may respond to ART, the experiences of pregnant and lactating women are likely to differ from those of the general population.

It is important to understand PLHIV's experiences with Treat‐all care to ensure programmes correspond to their needs, and can adequately and appropriately support them to engage with treatment and care to improve health outcomes 20, 21, 22. Additionally, from a public health perspective, suboptimal adherence among growing cohorts of asymptomatic patients could lead to drug resistance, which has been highlighted as a critical threat to eliminating AIDS by 2030 23, 24, 25, 26. This heightens the importance of understanding how treatment‐taking is experienced by asymptomatic PLHIV enrolled in Treat‐all care.

We examine asymptomatic PLHIV's experiences engaging with Treat‐all care in the Kingdom of Eswatini (formerly named Swaziland), including how treatment‐taking is navigated and motivated over the longer term (at least 12 months after initiation).

## 2 Methods

### 2.1 Study design

This paper draws on data that were collected between August 2016 and September 2017, within a longitudinal qualitative study on the experiences of asymptomatic PLHIV enrolled in chronic HIV care under the Treat‐all policy in Eswatini.

### 2.2 Study setting

This study took place in the Shiselweni region of Eswatini, where Médecins Sans Frontières (MSF) and the Ministry of Health (MoH) collaboratively provide decentralized HIV and tuberculosis care since 2007, with a Treat‐all implementation pilot beginning in October 2014. Eswatini has the highest reported HIV prevalence worldwide, estimated at 35% among women and 19% among men aged 15 to 49 years 27, with heterosexual sex being the main transmission route 28. Men are generally infected at older age than women, and HIV prevalence peaks at 54% among women aged 35 to 39 years and 49% among men aged 45 to 49 years 29.

The Treat‐all pilot project was implemented in the predominantly rural Nhlangano health zone, with eight primary healthcare clinics (largely rural, offering integrated HIV services) and one secondary health facility (urban, offering HIV care within a specific HIV‐related care department or as part of antenatal care). Patient enrolment to the pilot ended on 31 March 2016, with Treat‐all then becoming the standard of care in Nhlangano, and adopted into national policy in October 2016. Within the pilot project, prompt ART initiation was offered on the day of facility‐based HIV care registration. Forty‐nine per cent of patients initiated on the same day as enrolment to HIV care, and the majority of those who deferred initiated ART at a median of 10 days 30. Routine viral load monitoring was available at six months on ART and annually thereafter if results showed viral suppression. Communication of viral load results was prioritized for those with unsuppressed results, who were offered enhanced adherence counselling.

### 2.3 Participant recruitment

PLHIV participants were identified and recruited purposively to include only those recorded as newly diagnosed (within three months of enrolment to care) and considered clinically asymptomatic (WHO disease stage 1 and CD4 count ≥500 cells/mm^3^), using the Treat‐all patient database as a sampling frame. For the purposes of this analysis, we drew upon a subset of the study sample to include those who were enrolled at the beginning of the Treat‐all pilot, from October 2014 to June 2015, and who therefore would have been enrolled in at least 12 months at the time of first interview (see Table 1). This allowed examination of longer term, sustained engagement with treatment and care in the context of Treat‐all. We anticipated that recruiting young men (aged 16 to 25) would be challenging, as very few young men are infected with HIV in Eswatini, and as men often access treatment and care later in this setting.

**Table 1:** PLHIV participant characteristics (n = 17)^a^

Gender	
Female	9
Male	8
Age	
Young adult	
17 to 20 years	4 (all women)
21 to 25 years	3 (all women)^b^
Adult	
26 to 39 years	5 (1 woman, 4 men)
40 to 49 years	5 (1 woman, 4 men)
Enrolment to Treat‐all	
Oct 2014 to Dec 2014	5
Jan 2015 to Mar 2015	8
April 2015 to June 2015	4
Time between enrolment and initiation	
Same day	4
1 to 6 days	5
7 to 10 days	1
Ten days to one month	3
One to three months	2
Four to eight months	2
Treatment category	
On ART	10
Lost from treatment^c^	7

^a^Participant information relates to that recorded on the project patient database at the time of recruitment. ^b^Young adults (aged 16 to 25 years) and adults (aged 26 to 49 years) were purposively included in the sample. No young men were eligible due to the epidemiology of HIV in Swaziland meaning less young men are infected, and additionally men can access care later. ^c^Lost from treatment defined as those with a last recorded visit date of at least four months from the time of sample selection (to allow for those with three monthly refills).

Healthcare worker (HCW) participants were sampled purposively, to include those from all the nine clinics involved in the pilot, both MoH and MSF staff members, and a range of different treatment and care‐related positions, such as adherence counsellor (HIV‐positive peer supporters), nurse, nurse supervisor and doctor (Table 2).

**Table 2:** HCW participant characteristics (n = 20)

Position	
Adherence counsellor	5
Nurse/nurse supervisor	13
Doctor	1
Employer	
MoH	12
MSF	8
Facility	
Primary Health Clinic (8 clinics included)	17
Secondary Health Facility	3

HCW, healthcare worker; MSF, Médecins Sans Frontières; MoH, Ministry of Health.

Participants were recruited until data saturation was evidenced, that is, when adding further participants did not generate new findings relating to the particular topic or theme being investigated 31.

### 2.4 Data collection and analysis

Repeat in‐depth interviews with PLHIV participants aimed to gain insight into changes in participants’ accounts of their experiences with Treat‐all over time, and to build trust and rapport between interviewer and interviewee, enabling access to alternative layers of participants’ narratives beyond those participants may deem to be socially desirable. Interviews were based on topic guides and were primarily participant‐led, with first interviews focusing on the participant's life history, second their experiences of HIV testing and diagnosis, offer of treatment and experience starting ART. Subsequent interview(s) explored ongoing treatment‐taking, and revisited topics explored in earlier interviews to gain greater depth of insight and to explore any changes. Interviews were conducted from August 2016 to September 2017, the majority being held at participant homes, or at an alternative site if preferred.

Interviews with HCW participants explored views and experiences relating to Treat‐all implementation and providing treatment and care to asymptomatic patients. These were one‐time interviews conducted during February and March 2017, held in the clinics where HCWs worked.

Informed written consent was sought prior to all interviews, including for audio‐recording, which was re‐visited verbally at subsequent interviews for PLHIV participants. Interviews averaged 80 minutes, ranging from 50 minutes to 1 hour 40 minutes. Pseudonyms are used to protect participant confidentiality.

Detailed field notes were completed for each interview, and audio‐recordings were translated and transcribed. Data were analysed thematically using coding to identify patterns, categories and themes that emerged from participant accounts, drawing upon principles of grounded theory to raise findings to a conceptual level 32, 33. Initial codes were organized into a coding framework, forming the basis of continued analysis in Nvivo 11, which was developed and adapted as data collection and analysis progressed. Data collection and analysis followed an iterative process, enabling topic guides to be adapted to further probe emerging themes.

Ethics approval was obtained from the Eswatini Scientific and Ethics Committee, the London School of Hygiene and Tropical Medicine and MSF Ethics Review Boards prior to study commencement.

## 3 Results

### 3.1 Study participants

Selecting those enrolled to Treat‐all from October 2014 to June 2015 from the sample, seventeen PLHIV participants were eligible for inclusion in this analysis, including nine women and eight men, with fifteen interviewed three times, one interviewed four times and one interviewed twice. PLHIV participants had been on ART for a mean of twenty months at the time of the first interview, and there was a mean of eight months between the first and the last interview (see Table 1). Additionally, 20 HCW participants were interviewed once (see Table 2).

**Figure 1:**
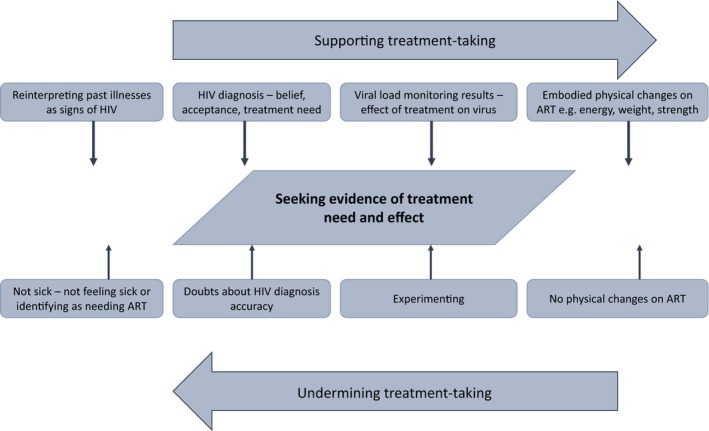
Diagram of findings relating to seeking evidence of treatment need and effect and their influence on treatment‐taking

Figure 1 depicts a summary of the study findings, which are elaborated as follows.

### 3.2 The perceived need for treatment influencing engagement with treatment and care

For PLHIV, perceiving need for treatment was important to motivate their taking it. For some, receiving an HIV diagnosis was itself a pointer to the need for treatment: “I had heard that if you are positive and do not take the treatment you could die…” (Nozipho, woman, 19 years). Although clinically asymptomatic, some participants described embodied signs of HIV which served to warn them of the potential risk of deteriorating health:

I started taking the treatment because I am afraid of getting sick and even at times I would have headache, and I would think probably it has started. (Sifiso, man, 41 years)

Some believed that treatment would prolong their life. This was particularly described by men living with HIV, with early initiation of treatment seen as enabling maintenance of strength and productivity, potentially reinforcing notions of masculine responsibility. Additionally, several PLHIV described experiencing physical changes after starting ART, such as increased energy and strength, increased weight and feeling healthier, for example no longer experiencing headaches. This reinforced a sense of value to the treatment and motivated continued treatment‐taking:

It is helping … because most of the time before I started taking the treatment I would feel that my body is going down, like when you wake up you'd find that the body is very tired most of the time. But then since I started the treatment I feel much better and healthier, and my body is energetic (Mandla, man, 36 years)

### 3.3 Doubts about treatment need and effect undermining treatment‐taking

However, not all participants perceived themselves as needing treatment or believed that it could benefit them. Several described doubts about the need for treatment in the absence of symptoms or signs of ill health:

The CD4 is high and it is just the positive word that is pointing, there are no other signs … I will take them when I can see that it [HIV] is now really there. (Zandile, woman, 23 years)

Some patients tell you that there is no need, they will start the treatment when they are sick, not now. (HCW15)

Those who did not experience any changes with ART appeared to doubt its effectiveness and the difference that treatment was making to them, which could undermine their motivation for taking it.

The problem I have is that I haven't seen the effectiveness of the treatment, because even when I go to the hospital the weight scale doesn't reflect much of a change … nothing changed because when I went there I wasn't sick, but I just went there healthy. (Jabulane, man, 31 years)

These doubts could also change over time, as participants sought evidence of the treatment's effectiveness and interpreted their own experiential changes as suggesting the treatment was working. For example, Jabulane, quoted from his second interview above, went on to interpret his health as improving by the third interview:

With the pills my brother, the way I see it, they are working because … I used to have flu every now and then … that is when I eventually got tested, but then since I started taking the treatment I no longer have such, I don't get influenza and go to the hospital.

Where doubts about treatment need and effectiveness were present, these appeared linked to PLHIV developing a sense of treatment fatigue over time:

I used to just get tired and I would sometimes skip some days and not go to collect them … there were times I just would be quiet and not take it, not because there is no food or something, I would just feel annoyed that why am I taking this treatment and what is it for? (Khanyisile, woman, 24 years)

HCW also described such doubts and treatment fatigue as a reason for some PLHIV disengaging from treatment and care:

After some years … you can find that a patient would say ‘ahh this treatment, I'm not sick anyway’ … so they believe they should stop the medication. (HCW10)

### 3.4 Seeking evidence of the treatment's effectiveness

Almost all PLHIV participants described wanting to see the difference that treatment was making to the virus and their health prognosis. Some also described a desire to hear more about their blood tests:

I want them to tell me is there something that it is doing, is there a difference. Even now I want them to tell me how my CD4 is doing, my CD4 was this much and now that I am continuing with the treatment, is it making any difference? (Celiwe, woman, 40 years)

For those who were informed of suppressed viral load results, this appeared to instil a belief in the effectiveness of treatment, which could be particularly important for those who initiated ART in the absence of symptoms, and which was said to motivate ongoing treatment‐taking: “[When told suppressed] it made me to feel free and to continue emphatically taking the treatment, because it means it is working.” (Vusi, man, 49 years)

The role of viral load monitoring as a potentially important tool for supporting adherence among patients who initiate ART when healthy under the Treat‐all policy was reiterated by HCW participants:

These patients are patients who came in healthy, they had no symptoms, so they are going to continue being healthy … they are not going to see what is better for them. So viral suppression is important … It would actually be good to have an initial baseline viral load and then you do monitoring following. (HCW19)

HCW recognized the importance of time explaining results:

I can admit and say we don't give that oomph for that time as equal as when the viral load is unsuppressed … yes we don't give that attention, maybe we can strengthen and say yes even if the patient's viral load is suppressed … CD4 count is high … we need to sit with the patient… and try to give the patient a lot of time and understanding. (HCW09)

Certain participants who were doubtful about the need for treatment and its effectiveness described experimenting with treatment‐taking, as a means of seeking such evidence:

There was a time … when I would think ‘haw this thing is not doing anything to me’ and then I stopped taking them and there was no sign of it being there … I never used to take them … I was not taking them well and nothing would happen and it would be the same as when I was taking them. (Nobuhle, woman, 17 years)

## 4 Discussion

This study investigated the experiences of asymptomatic PLHIV engaging with HIV care under the Treat‐all policy in Eswatini. Our findings suggest that it is important for PLHIV to have a sense of the difference treatment is making to them, in terms of its influence on their virus and health prognosis. Where PLHIV perceived need for treatment and believed it was effective in improving their health and potentially prolonging life, this motivated treatment‐taking and supported engagement with care. On the other hand, doubts relating to treatment need and effect could undermine engagement.

The biomedical logic framing Treat‐all assumes that individuals will adjust their actions once they are educated; however, treatment decisions surpass the biomedical realm 34. There can be dissonance between the biomedical rhetoric and lived experiences of those who engage with technologies 35, including in lay interpretations of what constitutes treatment necessity 36. In our study PLHIV could gain evidence of treatment effect through experiential, embodied changes with identifiable, physical improvements after being on ART, or through viral load monitoring results showing the impact of ART on the level of the virus. However, some PLHIV had doubts about the need for treatment and its effectiveness in the absence of any symptoms or signs of ill health, which could undermine their engagement and cause intermittent treatment‐taking. While others have examined the factors influencing ART initiation among asymptomatic PLHIV within Treat‐all 37, 38, 39, our study builds on these findings and provides insight into the influences on ongoing, longer term treatment taking, after an average of 20 months on ART.

Existing evidence from Eswatini suggests PLHIV can find it difficult to accept ART within Treat‐all when feeling healthy, with the belief that ART is for sick people 37, 40. Experiences with Option‐B+ programmes in Southern Africa have found some relate taking ART with being ill or having a low CD4 count, which can undermine asymptomatic pregnant and lactating women's engagement with treatment 19, 41. Pound *et al*. describe treatment‐taking as a concretization of illness, rather than health 42. As HIV treatment for preventative purposes within Treat‐all transforms HIV from an acute to chronic condition, the symptoms of disease and efficacy of treatment become less apparent 43. While some participants in our study felt they did not need treatment in the absence of symptoms, others reinterpreted past illnesses and physical experiences such as fatigue, low energy and weight loss as signs of HIV, which served to warn them of the potential imminent health deterioration without treatment, thereby motivating their engagement with care. Such reinterpretations of past events as indications of HIV were also found by Zhou, and influenced women's ART initiation within Option‐B+ 43, suggesting the importance of experiential, embodied experience in treatment decisions. Certain participants in our study felt that a HIV diagnosis itself pointed to the need for treatment. These findings therefore highlight the ways in which conceptions around health and treatment need are changing within the context of Treat‐all, and they may continue to do so as it becomes more commonplace.

Almost all participants in our study wanted to see the positive effect or difference treatment was making to them, seeking evidence of the treatment's effect either through their own embodied experiences or through biomarkers such as CD4 count and viral load, which served as indicators of their health status. Although some researchers have described the value placed on such indicators 44, our findings went further by suggesting that viral load monitoring can play an important role in supporting adherence to ART, offering patients a means to view the effect of treatment on the level of the virus. This may be of particular importance in Treat‐all contexts where PLHIV are increasingly initiating ART at earlier, asymptomatic phases of the disease. Routine viral load monitoring should be implemented universally as a tool to promote engagement in treatment and care, with communication of both suppressed results, as well as unsuppressed results, being emphasized.

Under previous treatment guidelines, recounting narratives of illness history and comparing pre‐ and post‐ART health could motivate continued treatment‐taking 12, 13. Although PLHIV are now increasingly initiating ART when clinically asymptomatic, findings from Option‐B+ suggest that interpretations of physical improvements on ART, in particular falling sick less often, feeling more energetic and therefore more productive can be important for supporting continued treatment‐taking 19, 43, 45. Certain PLHIV in our study who did not experience any such physical signs of HIV prior to starting ART, changes on ART or viral load results presented doubts about the need for treatment and its effectiveness. This could lead to treatment experimentation, where PLHIV could miss doses of treatment, seeking evidence of its effect. Conrad describes “non‐compliance” as a form of “self‐regulation,” where patients may alter the course of treatment to test its efficacy 46, as seen in mothers enrolled in Option‐B+ 43. This is particularly problematic as it poses risks for drug resistance 24, suggesting the importance of patients within Treat‐all starting treatment when they are ready, when they want treatment and exploring means of addressing their concerns about its effectiveness. PLHIV who had such doubts were also said to be more likely to develop a sense of treatment fatigue over time, where questioning the point of treatment undermines the energy for taking it. This could become increasingly relevant as Treat‐all patients are on treatment for longer periods of time.

### 4.1 Limitations

As the Treat‐all pilot was implemented by MSF and the MoH, patients may have received more support, including greater availability of viral load monitoring. However, the pilot aimed to examine the feasibility of Treat‐all under routine programmatic conditions, and the presence of viral load monitoring enabled unique insight into the supportive value of this tool for patients’ treatment‐taking. Though we were able to include PLHIV who had been enrolled in Treat‐all care for longer than previously reported, Treat‐all was still fairly new at the time of this study. It will be important to examine how these findings may change as Treat‐all becomes more commonplace, as well as to examine experiences of PLHIV with Treat‐all who have been on ART for longer.

## 5 Conclusion

This research highlights the importance of PLHIV perceiving need for treatment and having evidence of the benefits of their taking it, for motivating their ongoing, sustained treatment‐taking in the context of Treat‐all. Almost all participants described a desire for evidence of the need for treatment and its effect, with routine viral load monitoring potentially providing this. This could be particularly important for those who initiate ART when asymptomatic, who do not experience the transformative effects of ART and who can have doubts about the value of treatment, which potentially undermine treatment‐taking. It is important that programmes consider these findings to adapt patient support, to avoid the risk of PLHIV “experimenting” with treatment‐taking which could cause drug resistance to develop. This could include communicating to patients that there may be no notable difference in health status on initiating ART when asymptomatic, and that benefits of early ART include prolonging good health. Programmes should also ensure routine viral load monitoring is included as an integral component of HIV care within the Treat‐all policy, with a baseline viral load if possible, and ensuring suppressed results are communicated to patients.

## Competing interests

The authors declare that they have no competing interests.

## Authors’ contributions

SH was the principal investigator of the study, and with BK and AW conceived and designed the study. SH, ZT and VD collected and analysed data. AW also contributed to analysis and interpretation of data. SH wrote the first draft of the paper, and led on revisions with input from AW and JS. All authors commented on the paper and approved the final version.

## References

[1] World Health Organisation (WHO) . Guideline on when to start antiretroviral therapy and on pre‐exposure prophylaxis for HIV [Internet]. 2015 [cited 2015 Oct 28]. Available from: http://www.who.int/hiv/pub/guidelines/earlyrelease-arv/en/ 26598776

[2] Gardner EM , McLees MP , Steiner JF , Del Rio C , Burman WJ . The spectrum of engagement in HIV care and its relevance to test‐and‐treat strategies for prevention of HIV infection. Clin Infect Dis. 2011;52(6):793–800. 21367734 10.1093/cid/ciq243 PMC3106261

[3] Baggaley R , Doherty M , Ball A , Ford N , Hirnschall G . The strategic use of antiretrovirals to prevent HIV infection: a converging agenda. Clin Infect Dis. 2015;60 Suppl 3:S159–60. 25972496 10.1093/cid/civ091

[4] Granich R , Williams B , Montaner J . Fifteen million people on antiretroviral treatment by 2015: treatment as prevention. Curr Opin HIV AIDS. 2013;8(1):41–9. 23188178 10.1097/COH.0b013e32835b80dd

[5] Nachega JB , Uthman OA , del Rio C , Mugavero MJ , Rees H , Mills EJ . Addressing the Achilles’ heel in the HIV care continuum for the success of a test‐and‐treat strategy to achieve an AIDS‐free generation. Clin Infect Dis. 2014;59 Suppl 1:S21–7. 24926028 10.1093/cid/ciu299 PMC4141496

[6] Iwuji CC , Orne‐Gliemann J , Larmarange J , Okesola N , Tanser F , Thiebaut R , et al. Uptake of home‐based HIV testing, linkage to care, and community attitudes about ART in rural KwaZulu‐Natal, South Africa: descriptive results from the first phase of the ANRS 12249 TasP cluster‐randomised trial. PLoS Med. 2016;13(8):e1002107. 27504637 10.1371/journal.pmed.1002107 PMC4978506

[7] Iwuji CC , Orne‐Gliemann J , Larmarange J , Balestre E , Thiebaut R , Tanser F , et al. Universal test and treat and the HIV epidemic in rural South Africa: a phase 4, open‐label, community cluster randomised trial. Lancet HIV. 2018;5(3):e116–25. 29199100 10.1016/S2352-3018(17)30205-9

[8] Plazy M , El Farouki K , Iwuji C , Okesola N , Orne‐Gliemann J , Larmarange J , et al. Access to HIV care in the context of universal test and treat: challenges within the ANRS 12249 TasP cluster‐randomized trial in rural South Africa. J Int AIDS Soc. 2016;19:20913. 27258430 10.7448/IAS.19.1.20913 PMC4891946

[9] Vervoort SC , Borleffs JC , Hoepelman AI , Grypdonck MH . Adherence in antiretroviral therapy: a review of qualitative studies. AIDS. 2007;21(3):271–81. 17255734 10.1097/QAD.0b013e328011cb20

[10] Heestermans T , Browne JL , Aitken SC , Vervoort SC , Klipstein‐Grobusch K . Determinants of adherence to antiretroviral therapy among HIV‐positive adults in sub‐Saharan Africa: a systematic review. BMJ Glob Health. 2016;1(4):e000125. 10.1136/bmjgh-2016-000125 PMC5321378 28588979

[11] Tucker JD , Tso LS , Hall B , Ma Q , Beanland R , Best J , et al. Enhancing public health HIV interventions: a qualitative meta‐synthesis and systematic review of studies to improve linkage to care, adherence, and retention. Ebiomedicine. 2017;17:163–71. 28161401 10.1016/j.ebiom.2017.01.036 PMC5360566

[12] Bernays S , Seeley J , Rhodes T , Mupambireyi Z . What am I “living” with? Growing up with HIV in Uganda and Zimbabwe. Sociol Health Illn. 2015;37(2):270–83. 25421409 10.1111/1467-9566.12189

[13] Nam SL , Fielding K , Avalos A , Dickinson D , Gaolathe T , Geissler PW . The relationship of acceptance or denial of HIV‐status to antiretroviral adherence among adult HIV patients in urban Botswana. Soc Sci Med. 2008;67(11):1934. 10.1016/j.socscimed.2008.03.042 18455285

[14] Clouse K , Schwartz S , Van Rie A , Bassett J , Yende N , Pettifor A . “What they wanted was to give birth; nothing else”: barriers to retention in option B+ HIV care among postpartum women in South Africa. J Acquir Immune Defic Syndr. 2014;67(1):e12–8. 24977376 10.1097/QAI.0000000000000263 PMC6686681

[15] Knettel BA , Cichowitz C , Ngocho JS , Knippler ET , Chumba LN , Mmbaga BT , et al. Retention in HIV care during pregnancy and the postpartum period in the option B+ era: systematic review and meta‐analysis of studies in Africa. J Acquir Immune Defic Syndr. 2018;77(5):427–38. 29287029 10.1097/QAI.0000000000001616 PMC5844830

[16] Tenthani L , Haas AD , Tweya H , Jahn A , van Oosterhout JJ , Chimbwandira F , et al. Retention in care under universal antiretroviral therapy for HIV‐infected pregnant and breastfeeding women (‘Option B+’) in Malawi. AIDS. 2014;28(4):589–98. 24468999 10.1097/QAD.0000000000000143 PMC4009400

[17] Cataldo F , Chiwaula L , Nkhata M , van Lettow M , Kasende F , Rosenberg NE , et al. Exploring the experiences of women and health care workers in the context of PMTCT option B plus in Malawi. J Acquir Immune Defic Syndr. 2017;74(5):517–22. 28045712 10.1097/QAI.0000000000001273 PMC5340586

[18] McLean E , Renju J , Wamoyi J , Bukenya D , Ddaaki W , Church K , et al. “I wanted to safeguard the baby”: a qualitative study to understand the experiences of option B+ for pregnant women and the potential implications for “test‐and‐treat” in four sub‐Saharan African settings. Sex Transm Infect. 2017;93 Suppl 3:pii: e052972. 28736391 10.1136/sextrans-2016-052972 PMC5739848

[19] Katirayi L , Chouraya C , Kudiabor K , Mahdi MA , Kieffer MP , Moland KM , et al. Lessons learned from the PMTCT program in Swaziland: challenges with accepting lifelong ART for pregnant and lactating women – a qualitative study. BMC Public Health. 2016;16:1119. 27776495 10.1186/s12889-016-3767-5 PMC5078916

[20] INSIGHT START Study Group . Initiation of antiretroviral therapy in early asymptomatic HIV infection. N Engl J Med. 2015;373(9):795–807. 26192873 10.1056/NEJMoa1506816 PMC4569751

[21] The TEMPRANO ANRS 12136 Study Group . A trial of early antiretrovirals and isoniazid preventive therapy in Africa. N Engl J Med. 2015;373(9):808–22. 26193126 10.1056/NEJMoa1507198

[22] Song A , Liu X , Huang X , Meyers K , Oh D‐Y , Hou J , et al. From CD4‐based initiation to treating all HIV‐infected adults immediately: an evidence‐based meta‐analysis. Front Immunol. 2018;9:212. 29487595 10.3389/fimmu.2018.00212 PMC5816781

[23] World Health Organisation (WHO) . Guidelines on the public health response to pretreatment HIV drug resistance [Internet]. 2017 [cited 2018 Feb 14]. Available from: http://www.who.int/hiv/pub/guidelines/hivdr-guidelines-2017/en/

[24] World Health Organisation (WHO) . HIV Drug Resistance Report 2017 [Internet]. 2017 [cited 2018 Feb 14]. Available from: http://www.who.int/hiv/pub/drugresistance/hivdr-report-2017/en/

[25] Jena AB . Balancing disease eradication with the emergence of multidrug‐resistant HIV in test‐and‐treat policies. Clin Infect Dis. 2013;56(12):1797–9. 23487380 10.1093/cid/cit159

[26] Wagner BG , Blower S . Universal access to HIV treatment versus universal ‘Test and Treat’: transmission, drug resistance & treatment costs. PLoS ONE. 2012;7(9):e41212. 22957012 10.1371/journal.pone.0041212 PMC3434222

[27] UNAIDS . Country Factsheet Swaziland [Internet]. 2016 [cited 2018 Mar 20]. Available from: file:///C:/Users/lsh281543/Downloads/Country%20factsheets%20Swaziland%202016.pdf

[28] Swaziland Ministry of Health . Swaziland HIV Incidence Measurement Survey (SHIMS): First Findings Report; 2012.

[29] Swaziland Ministry of Health . Swaziland HIV incidence measurement survey 2: a population‐based HIV impact assessment [Internet]. 2017 [cited 2018 Oct 29]. Available from: https://phia.icap.columbia.edu/wp-content/uploads/2017/11/Swaziland_new.v8.pdf

[30] Kerschberger B , Nesbitt R , Mpala Q , Mamba C , Mabhena E , Kabore SM , et al. Feasibility of same‐day ART initiation under the WHO treat‐all approach in the public sector of Swaziland. Amsterdam: IAS; 2018.

[31] O'Reilly M , Parker N . “Unsatisfactory Saturation”: a critical exploration of the notion of saturated sample sizes in qualitative research. Qual Res. 2013;13(2):190–7.

[32] Bradley EH , Curry LA , Devers KJ . Qualitative data analysis for health services research: developing taxonomy, themes, and theory. Health Serv Res. 2007;42(4):1758–72. 17286625 10.1111/j.1475-6773.2006.00684.x PMC1955280

[33] Glaser BG . The future of grounded theory. Qual Health Res. 1999;9(6):836–45.

[34] Beckmann N . Responding to medical crises: AIDS treatment, responsibilisation and the logic of choice. Anthropol Med. 2013;20(2):160–74. 23898836 10.1080/13648470.2013.800805

[35] Young I , Flowers P , McDaid L . Can a pill prevent HIV? Negotiating the biomedicalisation of HIV prevention. Sociol Health Illn. 2016;38(3):411–25. 26498141 10.1111/1467-9566.12372 PMC5102670

[36] Kawuma R , Seeley J , Mupambireyi Z , Cowan F , Bernays S ; REALITY Trial Team . “Treatment is not yet necessary”: delays in seeking access to HIV treatment in Uganda and Zimbabwe. Afr J AIDS Res. 2018;17(3):217–25. 30132397 10.2989/16085906.2018.1490785

[37] Pell C , Vernooij E , Masilela N , Simelane N , Shabalala F , Reis R . False starts in “test and start”: a qualitative study of reasons for delayed antiretroviral therapy in Swaziland. Int Health. 2018;10(2):78–83. 29342259 10.1093/inthealth/ihx065

[38] Boyer S , Iwuji C , Gosset A , Protopopescu C , Okesola N , Plazy M , et al. Factors associated with antiretroviral treatment initiation amongst HIV‐positive individuals linked to care within a universal test and treat programme: early findings of the ANRS 12249 TasP trial in rural South Africa. AIDS Care. 2016;28 Suppl 3:39–51. 27421051 10.1080/09540121.2016.1164808 PMC5096681

[39] Mbonye M , Seeley J , Nalugya R , Kiwanuka T , Bagiire D , Mugyenyi M , et al. Test and treat: the early experiences in a clinic serving women at high risk of HIV infection in Kampala. AIDS Care. 2016;28:33–8. 27421050 10.1080/09540121.2016.1164804

[40] Adams AK , Zamberia AM . “I will take ARVs once my body deteriorates”: an analysis of Swazi men's perceptions and acceptability of Test and Start. Afr J AIDS Res. 2017;16:295–303. 29132279 10.2989/16085906.2017.1362015

[41] Kim MH , Zhou A , Mazenga A , Ahmed S , Markham C , Zomba G , et al. Why did I stop? Barriers and facilitators to uptake and adherence to ART in option B+ HIV care in Lilongwe, Malawi. PLoS ONE. 2016;11(2):e0149527. 26901563 10.1371/journal.pone.0149527 PMC4762691

[42] Pound P , Britten N , Morgan M , Yardley L , Pope C , Daker‐White G , et al. Resisting medicines: a synthesis of qualitative studies of medicine taking. Soc Sci Med 1982. 2005;61(1):133–55. 10.1016/j.socscimed.2004.11.063 15847968

[43] Zhou A . The uncertainty of treatment: women's use of HIV treatment as prevention in Malawi. Soc Sci Med. 1982;2016(158):52–60. 10.1016/j.socscimed.2016.04.013 27111435

[44] Renju J , Moshabela M , McLean E , Ddaaki W , Skovdal M , Odongo F , et al. ‘Side effects’ are ‘central effects’ that challenge retention in HIV treatment programmes in six sub‐Saharan African countries: a multicountry qualitative study. Sex Transm Infect. 2017;93 Suppl 3:e052971. 28736390 10.1136/sextrans-2016-052971 PMC5739838

[45] Ngarina M , Tarimo EAM , Naburi H , Kilewo C , Mwanyika‐Sando M , Chalamilla G , et al. Women's preferences regarding infant or maternal antiretroviral prophylaxis for prevention of mother‐to‐child transmission of HIV during breastfeeding and their views on Option B+ in Dar es Salaam, Tanzania. PLoS ONE. 2014;9(1):e85310. 24465532 10.1371/journal.pone.0085310 PMC3899007

[46] Conrad P . The meaning of medications: another look at compliance. Soc Sci Med 1982. 1985;20(1):29–37. 10.1016/0277-9536(85)90308-9 3975668

